# Supplementary material for: CODEX, a neural network approach to explore signaling dynamics landscapes
Source: Mol Syst Biol. 2021 Apr 9;17(4):e10026. doi: 10.15252/msb.202010026 (PMC8034356; doi:10.15252/msb.202010026)
Supplement: Supplementary file 1 — Appendix [file MSB-17-e10026-s002.pdf]

CODEX, a neural network approach to explore signaling dynamics landscapes

-

Appendix

Marc-Antoine Jacques[1], Maciej Dobrzyński[1], Paolo Armando Gagliardi[1], Raphael Sznitman[2], Olivier Pertz[1*]

[1] Institute of Cell Biology, University of Bern, Baltzerstrasse 4, 3012 Bern, Switzerland

[2] ARTORG Center for Biomedical Engineering Research, University of Bern, Murtenstrasse 55, 3008 Bern, Switzerland

*Correspondence and requests for materials should be addressed to O.P. (email: olivier.pertz@izb.unibe.ch).

Table of contents

Supplementary Figures

Appendix Figure S1……………………………………………………………………….3

Supplementary Notes

Appendix Note 1: Rationale for the choice of CNN architecture………………………..6

Appendix Note 2: CODEX creates features that isolates dynamics in synthetic data……………………………………………………………………………...7

Appendix Note 3: CODEX identifies TGFβ dose-dependent signaling states………...8

Appendix Note 4: CODEX identifies cell line-specific p53 responses under increasing ionizing radiation doses………………………………………………………..8

Appendix Note 5: CODEX identifies discriminative features in Drosophila Melanogaster speed movement data……………………………………………………...9

Bibliography……………………………………………………………………………………11

Supplementary Figures

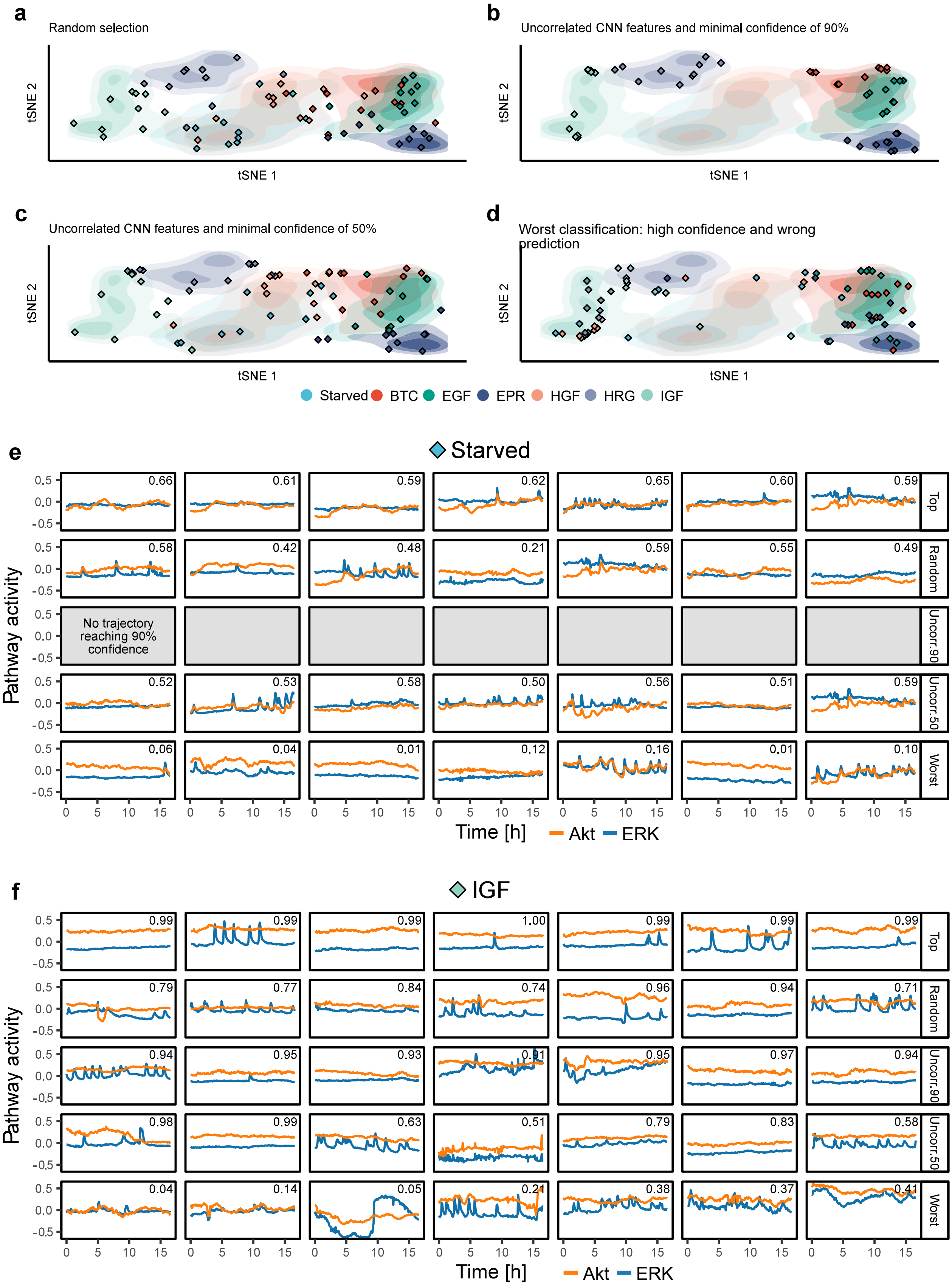

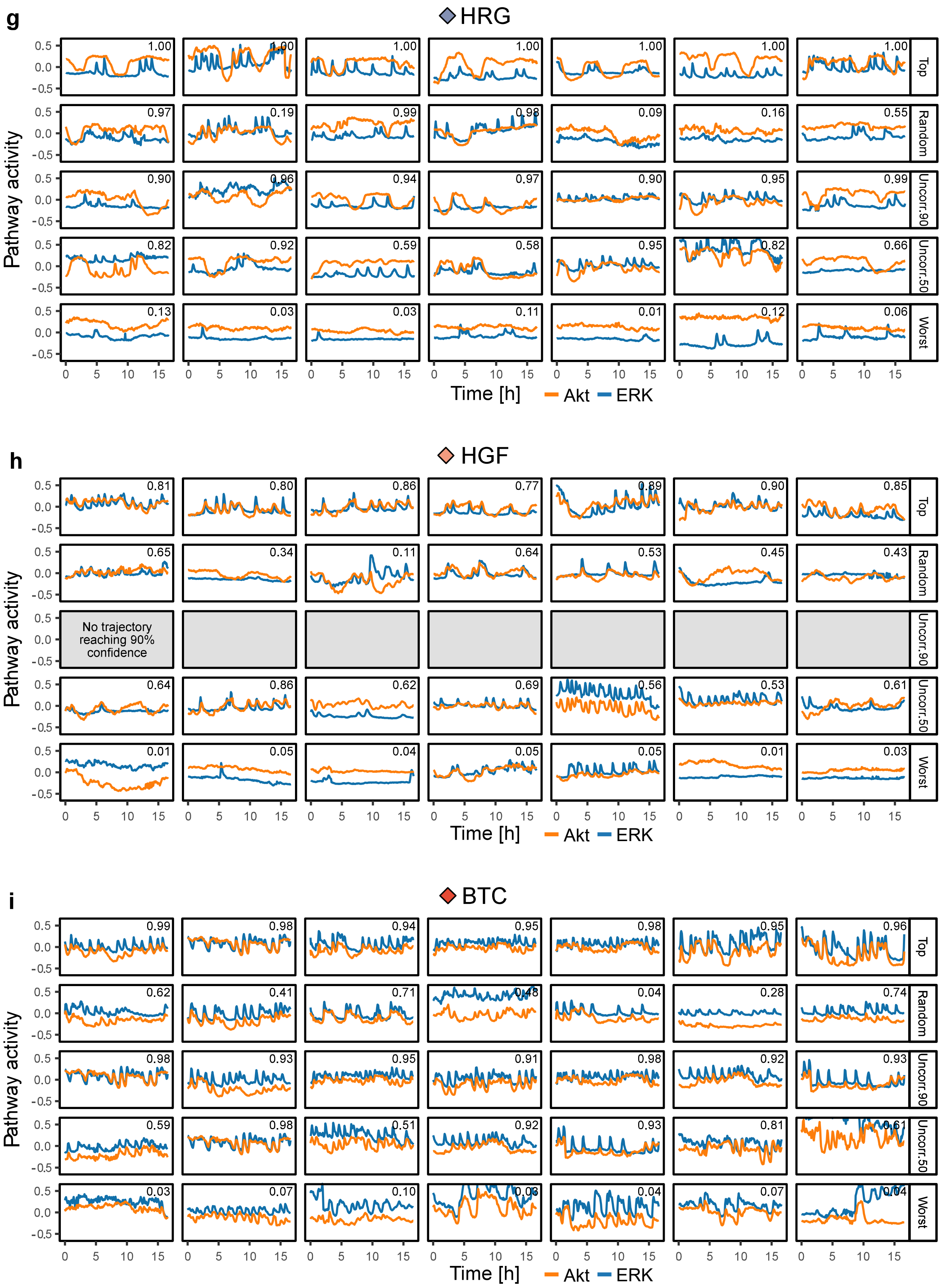

g
HRG
Pathway activity
0.5
0.0
-0.5
1.00 1.00 1.00 1.00 1.00 1.00 1.00
Top
0.97 0.19 0.99 0.98 0.09 0.16 0.55
Random
0.90 0.86 0.94 0.97 0.90 0.95 0.99
Uncorr.90
0.82 0.92 0.59 0.58 0.95 0.82 0.66
Uncorr.50
0.13 0.03 0.03 0.11 0.01 0.12 0.06
Worst
0 5 10 15
Time [h]
Akt
ERK
h
HGF
Pathway activity
0.81 0.80 0.86 0.77 0.89 0.90 0.85
Top
0.65 0.34 0.11 0.64 0.53 0.45 0.43
Random
No trajectory reaching 90% confidence
Uncorr.90
0.64 0.86 0.62 0.69 0.56 0.53 0.61
Uncorr.50
0.01 0.05 0.04 0.05 0.05 0.01 0.03
Worst
Time [h]
Akt
ERK
i
BTC
Pathway activity
0.99 0.98 0.94 0.95 0.98 0.95 0.96
Top
0.62 0.41 0.71 0.48 0.04 0.28 0.74
Random
0.98 0.93 0.95 0.91 0.98 0.92 0.93
Uncorr.90
0.59 0.98 0.51 0.92 0.93 0.81 0.61
Uncorr.50
0.03 0.07 0.10 0.03 0.04 0.07 0.04
Worst
Time [h]
Akt
ERK

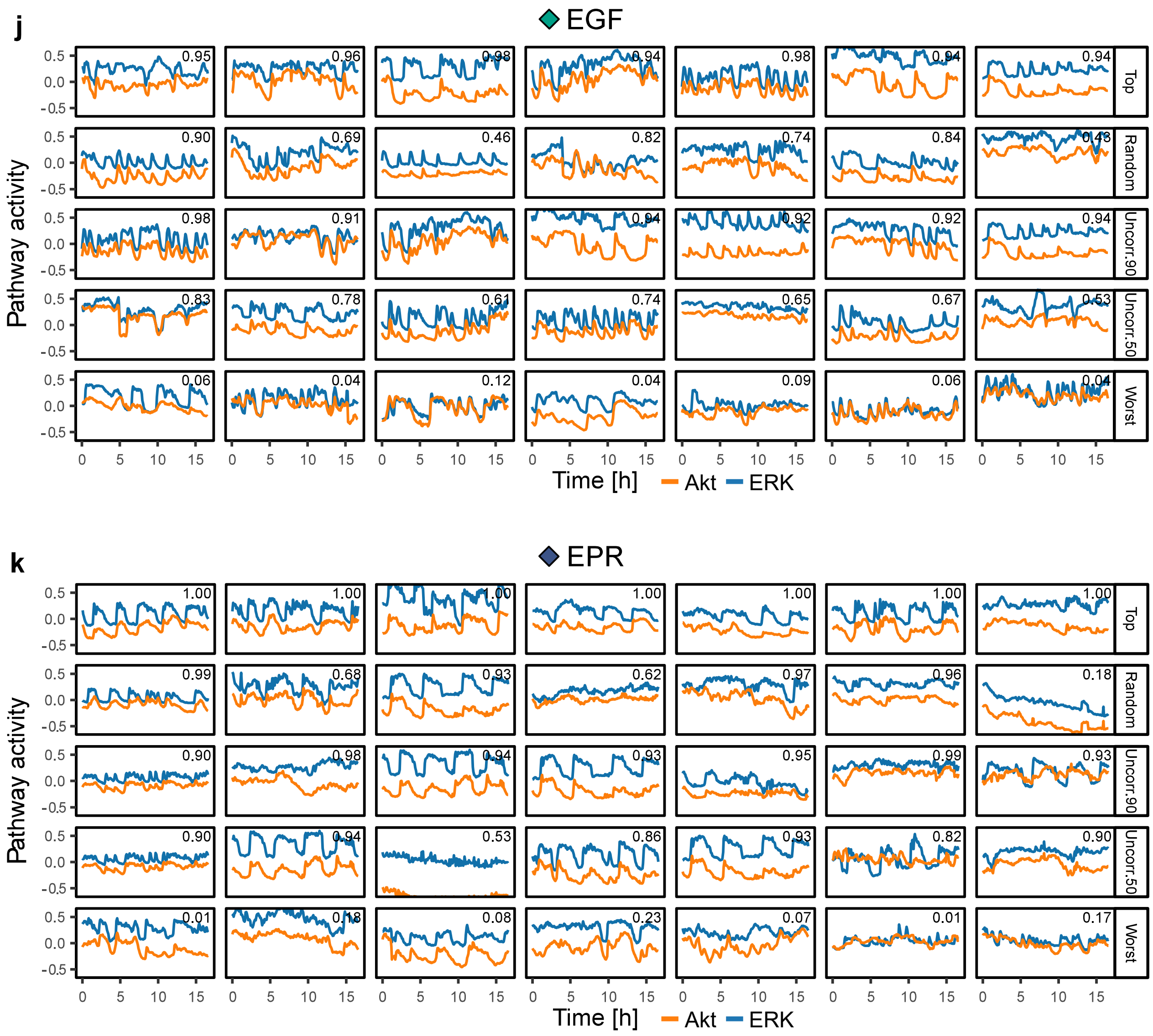

Appendix Figure S1: Comparison of prototype trajectories sampling strategies. a-d, Different strategies to sample prototype trajectories from the GF ERK/Akt dynamics dataset are presented on the same t-SNE projection of the CNN latent features of trajectories from the validation set. Prototype trajectories are indicated with diamonds, 10 trajectories are shown for each GF with each strategy. Missing diamonds in **b** or **c** indicate that none, or only a small proportion of trajectories reached the minimum confidence threshold. **a**, Random selection, **b**, Uncorrelated CNN features and minimal confidence of 90%, **c**, Uncorrelated CNN features and minimal confidence of 50%, **d**, Worst classification: high confidence and wrong prediction. **e-k**, Comparison of prototype trajectories identified using different sampling strategies. **e**, Starved, **f**, IGF, **g**, HRG, **h**, HGF, **i**, BTC, **j**, EGF, **k**, EPR.

Supplementary Notes

Appendix Note 1: Rationale for the choice of CNN architecture.

We encourage the use of a simple CNN architecture (Table EV1) that was previously reported in the literature, and that we also found to be a solid baseline through a wide range of datasets (Zhou *et al*, 2015; Wang *et al*, 2016). We believe that as little effort as possible should go into the network design and training, such that emphasis can be put on the interpretation of results. We thus favorized the ease of model training over predictive power, with smaller models that are easier and faster to train.

This CNN architecture also displays desirable aspects for our application. First, its plain feed-forward structure reduces the range of parameters to tweak if the classification performance is not satisfactory with the defaults. Optimizing a CNN architecture is a very time-consuming activity that requires expertise and, despite recent advances(Springenberg *et al*, 2016; Frankle & Carbin, 2018), still involves a lot of trials and errors. Reducing the time spent on this step is essential to quickly start with mining the results. Second, the reduced number of parameters enables fast training, even on consumer-grade GPU, and the use of smaller training datasets. The latter implies that training a CNN from scratch is realistic even with relatively small datasets that are usually obtained in single-cell biology. This is an important requirement in cases where no similar and large annotated datasets can be used for transfer learning. Third, a strong countermeasure to overfitting is embedded in this architecture, in the form of a global average pooling (GAP) layer. Not only does GAP help to obtain a reasonable model, the introduction of a bottleneck in the size of the latent features is also useful to project them in low dimensions without large distortions. [PAG2] Finally, this architecture is compatible with the creation of CAMs which we use to identify characteristic motifs in the trajectories.

Though the exact architecture of the CNN is flexible, the CAM motif mining approach is restraining the choice of architectures because it explicitly requires the use of global average pooling (Zhou *et al*, 2015). This limitation can however be circumvented by replacing CAM with another technique that creates saliency maps such as: grad-CAM, a generalization of CAM which is compatible with more architectures (Selvaraju *et al*, 2017) or guided backpropagation, another popular choice (Springenberg *et al*, 2014).

As a general guideline to start training such a model, we recommend to keep the number of CNN features, L2 penalty and the initial learning rate at their default value and to focus on adjusting the learning rate decrease schedule. If the model underfits after a few trials, try

increasing the number of features. If the model overfits, try to decrease the number of features and/or to increase the L2 penalty.

Appendix Note 2: CODEX creates features that isolates dynamics in synthetic data.

We first evaluated the ability of a supervised CNN classifier to classify the dynamic landscape in a synthetic time-series dataset, even if this separation is not necessary to perform the classification task perfectly. To do so, we created a synthetic dataset resembling pulsatile signaling activities and trained a CNN to recognize the different synthetic classes (Fig.EV1). All trajectories comprise 4 peak events which can be either full Gaussian peaks or half truncated ones. The dataset comprises 2 classes which differ by their number of full peaks. Trajectories from the first class comprise 0, 1 or 2 full peaks, while trajectories in the second class comprise 2, 3 or 4 full peaks (see Methods). Because the abundance of full peaks is the only difference in the process that generates these curves, there is an ambiguous case when an input trajectory comprises 2 full peaks. Hence the theoretical maximum accuracy for this classification task is 80%, a performance that the CNN reached after a few epochs of training.

The projection of the CNN features learnt for this task shows well-separated trajectory clusters which are grouping together trajectories with a common number of full peaks (Fig.EV1a, b). The trajectories for which the model was most confused between the 2 classes (“Low confidence”) all harbored 2 full peaks, hence were sampled from the only ambiguous case. This empirical observation is interesting because the model is only optimized to minimize the classification loss, such that the clusters containing 0 or 1 full peak and the clusters containing 3 and 4 full peaks could be merged together without affecting the classification performance. This illustration exposes the core intuition behind CODEX, that CNN features can naturally evolve to capture shapes in the data even without hard constraints.

Further, we verified that the motifs captured by CAMs (see Methods) were cleanly isolating class-discriminative motifs (Fig.EV1c, d). As expected, these motifs contained the tips of the peaks which are either full or truncated, but no flat part of the trajectories which are common to both classes. This also shows that both symmetries of truncation are captured. Finally, one can notice a bias towards the right over the left truncation since cluster 2 is less represented than cluster 3 despite being equally abundant in the trajectories. This last point indicates that the abundance of CAM clusters should be taken with caution. The possible sources of variation are the model training itself as well as the selection of trajectories from which to extract the motifs.

Appendix Note 3: CODEX identifies TGFβ dose-dependent signaling states.

To further showcase CODEX, we used it to analyze a dataset in which SMAD2 activity is measured in response to a dose challenge of TGFβ in MCF10A cells (Strasen *et al*, 2018). In this study, a fusion reporter between SMAD2 and YFP provides a readout for SMAD2 activity by computing the relative abundance of the reporter between the nucleus and the cytoplasm. We trained a CNN to recognize the doses of TGFβ that were given to the cells based on their SMAD2 trajectories.

In the original study, shape-based clustering of single-cell trajectories using DTW revealed a continuum of SMAD2 heterogeneous signaling states in response to the TGFβ dose response. We used this already published DTW-based clustering as a global indication of the shape of each trajectory. We then compared how the CNN features for each trajectory were arranged with respect to both the TGFβ dose and the DTW clusters. The projection of the CNN features revealed some entanglements between the TGFβ doses which matched the heterogeneity of SMAD2 signaling at all stimulation doses (Fig.4a). Interestingly, this entanglement seemed largely smoothed out when comparing the CNN features to the DTW clusters (Fig.4b). This again hints that despite the CNN features being learnt with the objective to separate the input classes, they still evolve to capture dynamic trends of the data.

From this observation we hypothesized that directly clustering the CNN features could also provide classes of representative dynamics in the data, similarly to what is done by DTW clustering (Fig.4c). We found the resulting clusters to be in slightly better agreement with the DTW clusters than with the TGFβ doses (Fig.4d, e). To summarize, we found that the CNN features clusters also efficiently captured trajectory profiles (Fig.4f). Visual inspection of representative trajectories suggests that the CNN features performed slightly better in separating flat from weak responders in comparison with the DTW clusters (CNN features clusters 1 and 2; DTW cluster 1).

Appendix Note 4: CODEX identifies cell line-specific p53 responses under increasing ionizing radiation doses.

To further showcase CODEX, we used it to analyze a much larger dataset with more classes. We used a study in which p53 activity dynamics was reported in 12 cancer cell lines under exposure to 5 different doses of ionizing radiation, yielding a total of 60 classes (Stewart-Ornstein & Lahav, 2017). p53 abundance was reported with a live-cell reporter consisting of a p53-YFP fusion construct.

After training, the projection of the CNN features shows that cell lines, irrespectively of the radiation dose, tend to have trajectories with similar features (Fig.5a, b). This strongly suggests that the cell line, rather than the radiation dose, is the major factor of p53 response variability in this dataset.

We then evaluated whether CODEX could recapitulate cell line-specific behaviors that were previously reported. To do so, we clustered the trajectories on the base of their CNN features into 6 clusters (Fig.5c). Similarly to what we observed for the TGFβ/SMAD2 dataset (Fig.4f), we found that the CNN features clusters capture identifiable trajectory shapes (Fig.5d): sustained activity for cluster 1 and 2, flat baseline for cluster 3, oscillations of large amplitude for cluster 4, oscillations of small amplitude for cluster 5 and a single pulse of activity for cluster 6. We then reported the distributions of these different clusters through the combinations of cell lines and radiation doses (Fig.5e, f). We found similar results to the original study, in which curated features were used to discriminate among dynamic signaling states. For example, MCF7 trajectories strikingly switch from a single pulse (cluster 6) to an oscillatory regime (cluster 5) with increasing radiation doses. Similarly, U2OS cells switched from an oscillatory regime (cluster 5) to a more sustained one (clusters 1 and 2) at high radiation doses. Other cell lines, on the contrary, adopted a consistent behavior through all radiation doses such as HCT116 cells which only display a single pulse of p53 activity (cluster 6) or MALME3 cells which display only low amplitude responses (clusters 3 and 6). CODEX could therefore recapitulate important findings in a large time-series dataset with very little human input and in about one hour for training the model.

Appendix Note 5: CODEX identifies discriminative features in *Drosophila Melanogaster* speed movement data.

We then tested if CODEX can be generalized to completely different time-series datasets. For this purpose, we applied CODEX to a univariate time-series dataset that describes the movement speed of *Drosophila* motility in a tube over 12 hours (Fulcher & Jones, 2017). There are 4 classes in this dataset that correspond to the combination of the *Drosophila* sex and whether the observation is carried under day or night light conditions. The trajectories are very long and show a strong alternance between resting phases and extreme events that are very different from the smooth series in the signaling datasets. Despite this, using the same CNN architecture, the trained model showed excellent prediction capabilities (accuracy of 90%) that are similar to the ones obtained in the original study with a classifier that uses hundreds of classical time-series features (accuracy of 95%). T-SNE projection of the CNN features clearly

separated the 4 classes based on *Drosophila* motility trajectories, and identified prototype trajectories (Fig EV5a,b).

We then investigated whether a correspondence between CODEX's output and the classical, interpretable features could be found. We observed that the criteria that separates male from female behaviors were retrieved in the CNN predictions. In the previous study, two of the main reported differences between males and females' movements are that females present a lower autocorrelation structure and a higher spectral flatness in their movement. We could retrieve this trend in the data and observed that the top prototype trajectories for female classes had a significantly lower autocorrelation and higher spectral flatness than their non-prototype counterparts (Fig.EV5c). More generally, we observed that a lower autocorrelation structure and a higher spectral flatness correlated with increased prediction odds for female classes (Fig.EV5d). However, the significant sampling of extreme individuals, regarding both features, was not observed for male prototypes. One could have expected male trajectory prototypes to also be significantly different from the other features but with the reverse effect than females. This could be due to the fact that the CNN was trained to separate all combinations of sex and night and not only the *Drosophila* sex. This absence of trend calls for caution when making a parallel between the CNN predictions and interpretable features from another set, especially if the former was trained on a different grouping than the one of interest.

Bibliography

Frankle J & Carbin M (2018) The Lottery Ticket Hypothesis: Finding Sparse, Trainable Neural Networks. *arXiv:1803.03635 [cs]*

Fulcher BD & Jones NS (2017) hctsa: A Computational Framework for Automated Time-Series Phenotyping Using Massive Feature Extraction. *Cell Syst.* **5:** 527–531.e3

Selvaraju RR, Cogswell M, Das A, Vedantam R, Parikh D & Batra D (2017) Grad-CAM: Visual explanations from deep networks via gradient-based localization. In *Proceedings of 2017 IEEE International Conference on Computer Vision (ICCV)* pp 618–626. IEEE

Springenberg JT, Dosovitskiy A, Brox T & Riedmiller M (2014) Striving for Simplicity: The All Convolutional Net. *arXiv preprint*

Springenberg JT, Klein A, Falkner S & Hutter F (2016) Bayesian optimization with robust Bayesian neural networks. In *Advances in Neural Information Processing Systems* pp 4134–4142.

Stewart-Ornstein J & Lahav G (2017) p53 dynamics in response to DNA damage vary across cell lines and are shaped by efficiency of DNA repair and activity of the kinase ATM. *Sci. Signal.* **10:**

Strasen J, Sarma U, Jentsch M, Bohn S, Sheng C, Horbelt D, Knaus P, Legewie S & Loewer A (2018) Cell-specific responses to the cytokine TGFβ are determined by variability in protein levels. *Mol. Syst. Biol.* **14:** e7733

Wang Z, Yan W & Oates T (2016) Time Series Classification from Scratch with Deep Neural Networks: A Strong Baseline. *arXiv:1611.06455 [cs, stat]*

Zhou B, Khosla A, Lapedriza A, Oliva A & Torralba A (2015) Learning Deep Features for Discriminative Localization. *arXiv:1512.04150 [cs]*
