# Supplementary material for: CODEX, a neural network approach to explore signaling dynamics landscapes
Source: Mol Syst Biol. 2021 Apr 9;17(4):e10026. doi: 10.15252/msb.202010026 (PMC8034356; doi:10.15252/msb.202010026)
Supplement: Supplementary file 2 — Expanded View Figures PDF [file MSB-17-e10026-s004.pdf]

# Expanded View Figures

**Figure EV1. CNN highlights discriminative dynamics motifs in synthetic data.**

A A synthetic dataset resembling pulsatile signaling activities was created, and a CNN was trained to recognize its two classes (see Materials and Methods and Appendix Note 2). Each trajectory comprises four pulsing events that consist of either full Gaussian peaks or half-truncated Gaussian peaks. Events are triggered at random locations but with a minimal distance from each other. The class labeled “Full” displays predominantly full peaks (2, 3, or 4), while the class labeled “Truncated” displays predominantly truncated Gaussian peaks (0, 1, or 2 full peaks).

B t-SNE projection of the CNN features of the trajectories in the validation set. Each cluster of points regroups trajectories based on their number of full peaks. Diamonds indicate prototype trajectories.

C Prototype trajectories (blue and red) sampled by selecting trajectories whose CNN features were uncorrelated (see Materials and Methods), and trajectories for which the model confidence is lowest (green). Trajectories are the ones indicated with diamonds in (B). The number of full peaks in each trajectory is indicated next to the diamond symbol. The minimal threshold of model confidence to sample prototypes was set to 90%.

D Motifs extracted with the CAM method (see Materials and Methods). Motifs were clustered with DTW distance and Ward linkage. Representative medoid motifs of the clusters are shown. The class of the trajectory from which the motif was extracted is indicated in the top-right corner.

E Distribution of the motifs in the clusters according to the class of their origin trajectory.

© 2021 The Authors

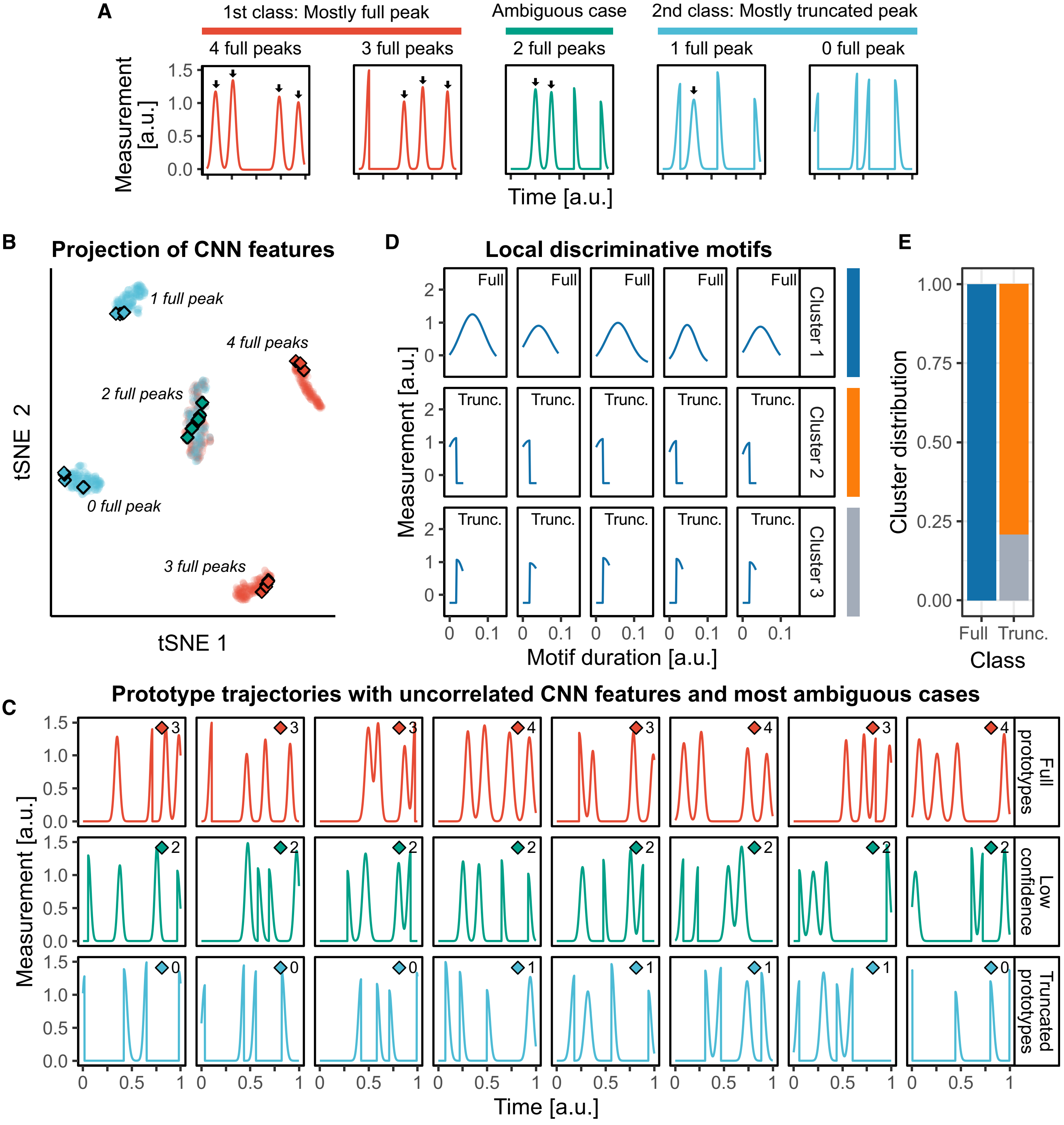


**Figure EV1.**

 © 2021 The Authors

**Figure EV2. Fluorescent biosensors of ERK and Akt activity report single-cell signaling dynamics in response to growth factors.**

A Scheme of the multiplexed, genetically encoded ERK (ERK-KTR-mTurquoise2) and Akt (FoxO3A-mNeonGreen) biosensors and the constitutive nuclear marker (H2B-miRFP703). Both biosensors translocate from nucleus to cytosol when phosphorylated by their respective kinase.

B Image analysis pipeline to obtain single-cell ERK/Akt trajectories. A random forest classifier (Ilastik) was trained to segment nuclei. Nuclei (red outline) are used for tracking each cell across the time lapse. Expansion of the nuclear mask provides a cytosol area (cyan outline). The ratio of pixel intensities of the cytosolic over the nuclear area (C/N) provides a proxy for ERK or Akt activity, which is displayed over each nucleus with a specific color code (high/low ERK/Akt activity = warm/cold colors, respectively).

C ERK and Akt activity channels in MCF10A monolayers 25 h after the stimulation with the different growth factors.

D Randomly selected ERK and Akt activity single-cell trajectories from MCF10A monolayers treated with the different growth factors. The dashed black vertical line represents the first frame of the $2^{nd}$ phase of signaling trajectories used to train CNN. GFs have been added at the beginning of the traces.

E Population averages of ERK/Akt activities of a whole field of view. Shading indicates 95% confidence interval of the mean. At least 1,200 cells for each GF pooled from two technical replicates.

© 2021 The Authors

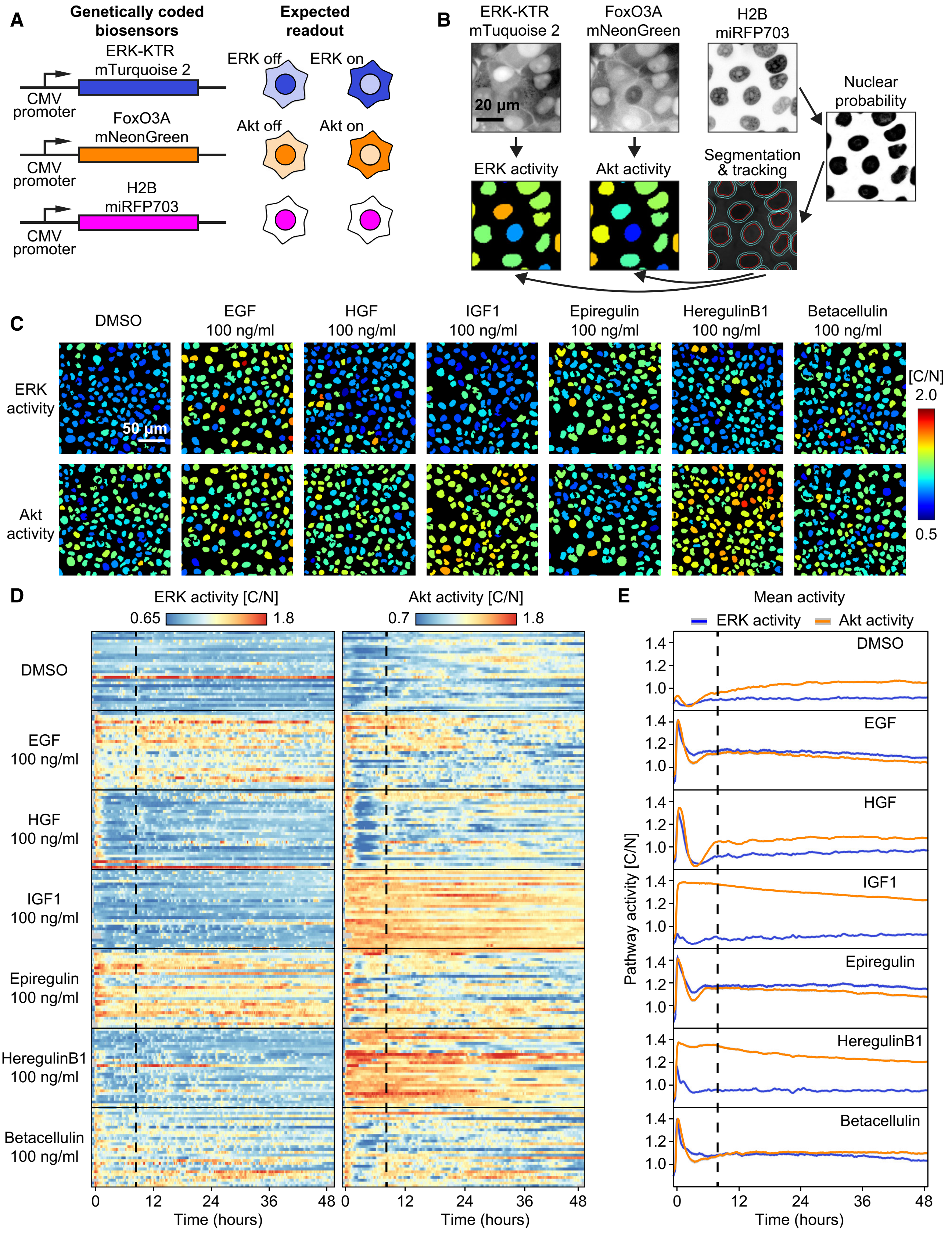


**Figure EV2.**

 © 2021 The Authors

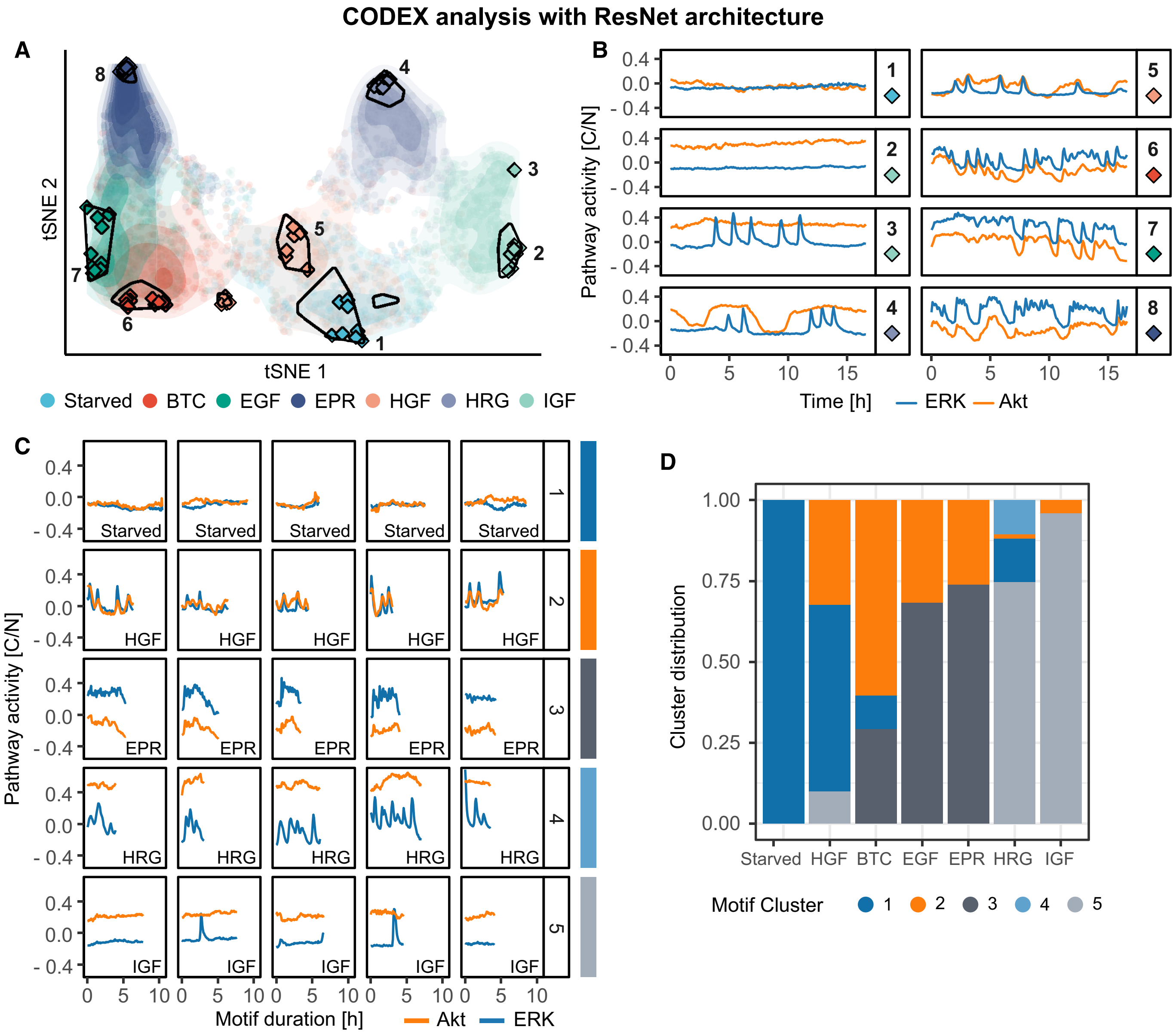


**Figure EV3. Applying CODEX with a ResNet architecture yields similar results to the ones obtained with a plain CNN architecture.**

A t-SNE projection of the ResNet features of all ERK/Akt trajectories in the validation set. Each point represents a bivariate ERK/Akt trajectory from a single cell. Hulls indicate areas associated with a strong classification confidence for each GF. Shading shows the point densities. Diamonds indicate the positions of the 10 top prototypes (see Materials and Methods) for each GF.

B Representative ERK/Akt prototype trajectories of the area indicated in (A). Each pathway activity was preprocessed by removing the average activity of this pathway across all trajectories in the training set.

C Discriminative signaling motifs were extracted from the training and validation top prototypes using a CAM-based approach (see Materials and Methods). The motifs were clustered using DTW distance and squared Ward's linkage. Representative motifs of each cluster, based on the minimization of mean intra-cluster distance, are displayed. Bottom-right labels indicate the class of the trajectory from which the motif was extracted. Each pathway activity was preprocessed by removing the average activity of this pathway across all trajectories in the training set.

D Distribution of the signaling motifs clusters across the GF treatments.

© 2021 The Authors

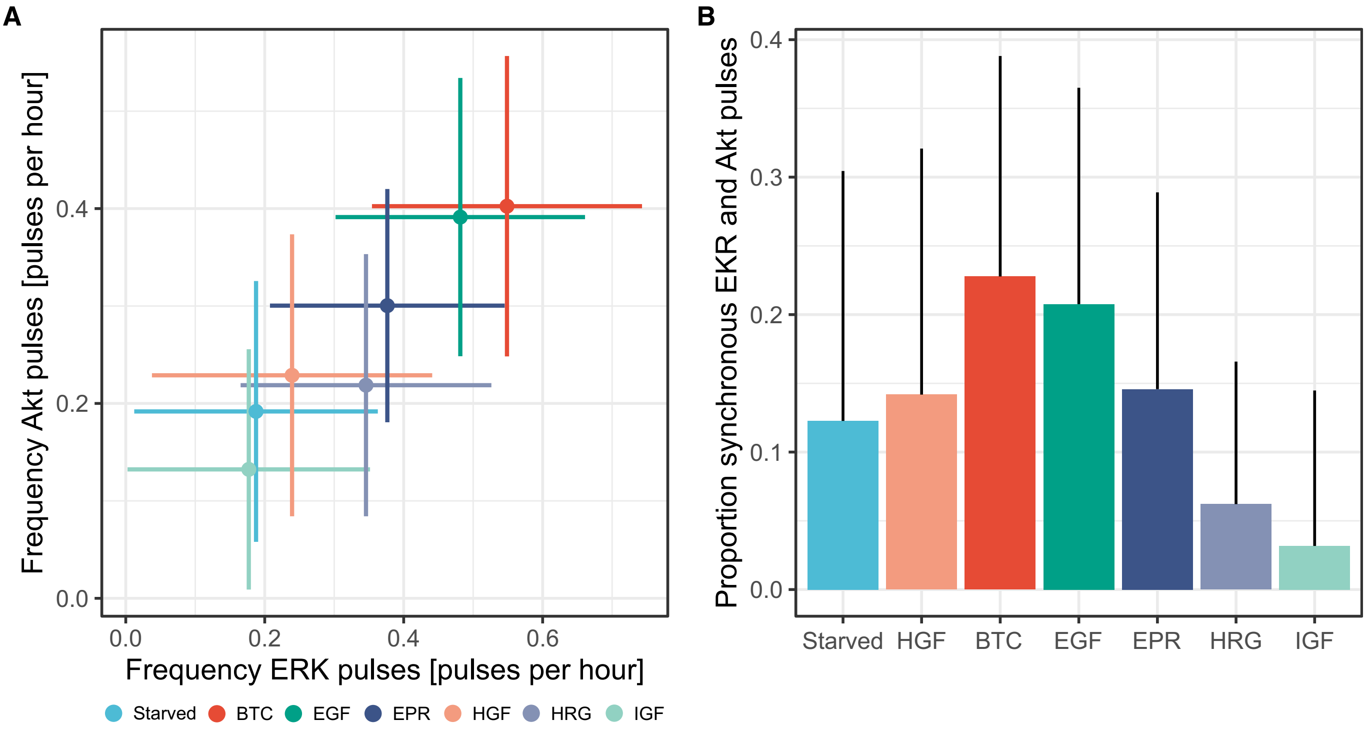


**Figure EV4. Quantification of additional interpretable, class-discriminative features identified by visual inspection of the CODEX output.**

A Scatter plot of frequency of ERK peaks versus frequency of Akt peaks in single cells. Crosses indicate the mean values and the standard deviations. At least 1,200 cells for each GF pooled from two technical replicates. Colors indicate the GF and are the same as in (B).

B Proportion of synchronous ERK and Akt peaks in single cells. Mean and standard deviation are shown. At least 1,200 cells for each GF pooled from two technical replicates.

 © 2021 The Authors

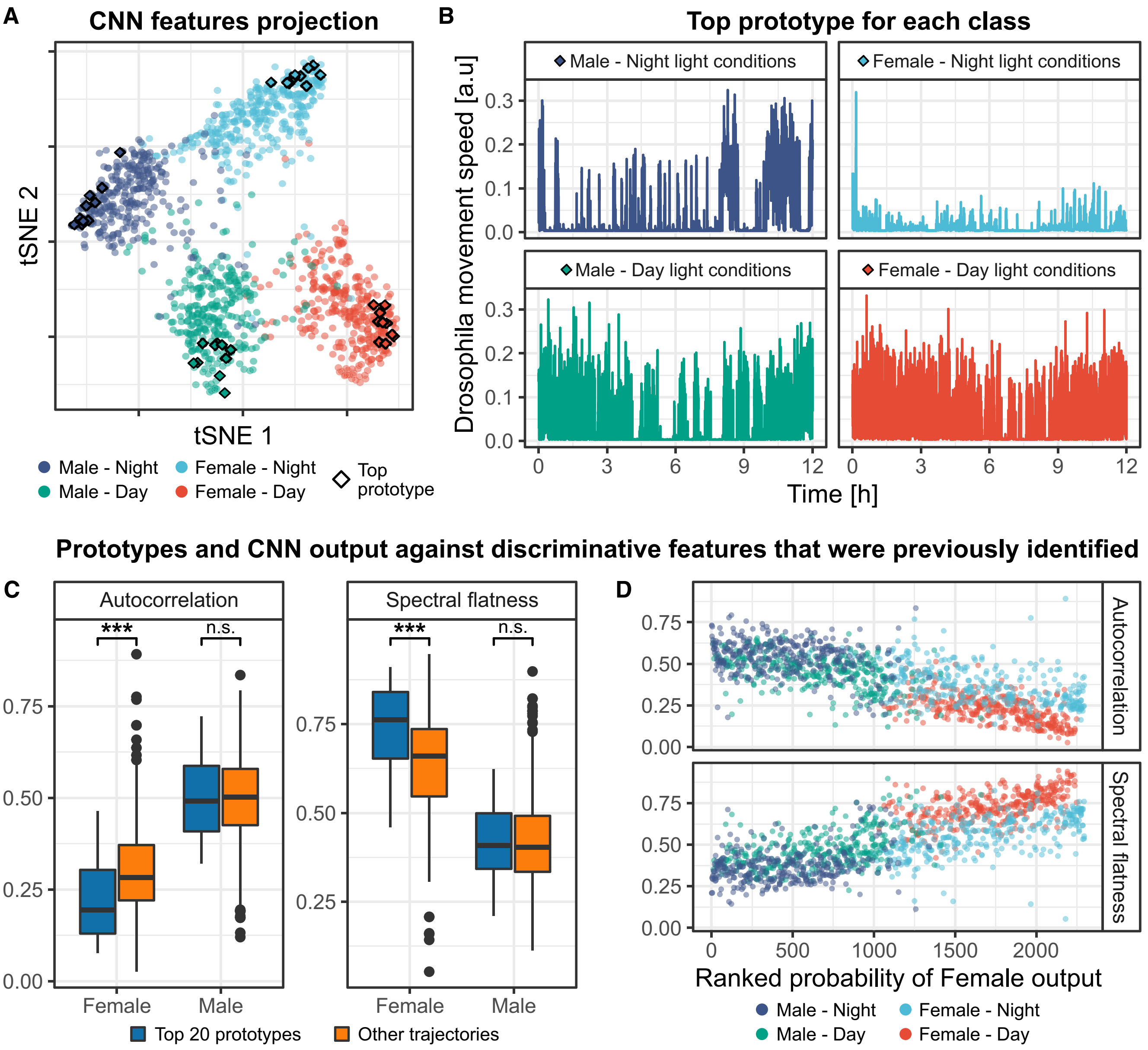


**Figure EV5. CODEX identifies different sex- and light-dependent Drosophila movements.**

Male and female Drosophila movements in a tube were recorded over 12 h, in presence or absence of light, and reported as univariate time series (Fulcher & Jones, 2017). A CNN was trained to recognize the combination of sex and light conditions based on the individual movements (see Materials and Methods and Appendix Note 5).

A t-SNE projection of the CNN features for the training, validation, and test sets pooled together. Diamonds indicate the 10 top prototypes for each class.

B The top prototype trajectory for each class.

C Comparison of autocorrelation levels and spectral flatness between the 20 top female prototypes and 20 top male prototypes against non-prototype trajectories. Female (resp. male) prototypes were obtained by pooling the 10 top prototypes of female (resp. male) drosophila under both day and night light conditions. Boxes indicate the upper and lower quartiles, the central band indicates the median and whiskers extend to individuals up to 1.5 interquartile away from the median. Medians of the distributions were compared with two-sided Wilcoxon's tests; ****P*-value $< 0.01$. $n = 574$ for non-prototype female trajectories; $n = 524$ for non-prototype male trajectories.

D Autocorrelation level and spectral flatness against the probability of trajectories to be classified as a female class. The latter probability is the sum of the probability of trajectories to belong to the female-day and female-night classes. The resulting sum was ranked, such that rank 1 corresponds to the probability of the trajectory that has the lowest prediction outcome to be classified as female. Autocorrelation levels were computed by averaging autocorrelation values for lags ranging from 1 to 10 time points.

© 2021 The Authors
